# Mutators and long-term molecular evolution of pathogenic Escherichia coli O157:H7.

**DOI:** 10.3201/eid0404.980411

**Published:** 1998

**Authors:** T S Whittam, S D Reid, R K Selander

**Affiliations:** Institute of Molecular Evolutionary Genetics, Pennsylvania State University, University Park 16802, USA. tsw1@psu.edu

## Abstract

It has been proposed that an increased mutation rate (indicated by the frequency of hypermutable isolates) has facilitated the emergence of Escherichia coli O157:H7. Analysis of the divergence of 12 genes shows no evidence that the pathogen has undergone an unusually high rate of mutation and molecular evolution.


### Mutators and Long-Term Molecular Evolution of Pathogenic *Escherichia coli* O157:H7

**Thomas S. Whittam, Sean D. Reid, and Robert K. Selander**
Pennsylvania State University, University Park, Pennsylvania, USA

It has been proposed that an increased mutation rate (indicated by the frequency of hypermutable isolates) has facilitated the emergence of *Escherichia coli* O157:H7. Analysis of the divergence of 12 genes shows no evidence that the pathogen has undergone an unusually high rate of mutation and molecular evolution.

*Escherichia coli* O157:H7, a highly virulent organism first linked to infectious disease in 1982 (1) and now found worldwide, has caused serious foodborne epidemics in the United States, Japan, and Europe (2). One hypothesis for the emergence and rapid spread of this organism is that strong mutator alleles enhance genetic variability and accelerate adaptive evolution (3). LeClerc et al. (3) found that more than 1% of O157:H7 strains had spontaneous rates of mutation that were 1,000-fold higher than those of typical *E. coli.* These mutator strains were defective in methyl-directed mismatch repair (MMR) as a result of deletions in the intergenic region between the *mutS* and *rpoS* genes (3). According to the mutator hypothesis, a pathogen able to enter a transient hypermutable state could overcome the fitness costs of deleterious mutations by accruing new genetic variation at times critical for survival and colonization of new hosts.

Adaptive evolution by transient or prolonged states of hypermutation can cause neutral mutations to rapidly accumulate throughout the genome. To detect possible elevation in the rate of molecular evolution in the emergence of *E. coli* O157:H7, we compared 12 genes with housekeeping functions (Figure) that have been sequenced in both *E. coli* O157:H7 and *E. coli* K-12 (a commensal organism), as well as in an outgroup species, *Salmonella enterica* serotype Typhimurium. The evolutionary distance (expressed in point mutations per 100 sites) between Typhimurium and K-12 is shown against the distance between Typhimurium and O157:H7 for synonymous and nonsynonymous sites separately (Figure). The line indicates equal rates of evolution in the two

Address for correspondence: Thomas S. Whittam, Institute of Molecular Evolutionary Genetics, Pennsylvania State University, University Park, PA 16802, USA; fax: 814-865-9131; e-mail: tsw1@psu.edu.

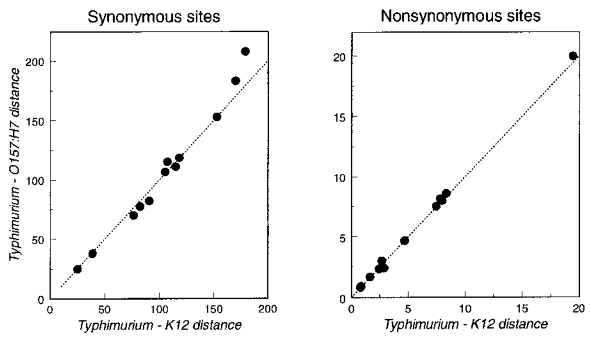

Figure. Evolutionary distance in terms of synonymous and nonsynonymous changes per 100 sites (4) for 12 genes sequenced from *Escherichia coli* O157:H7, *E. coli* K-12, and *Salmonella enterica* Typhimurium. The points for synonymous sites are (left to right): *gap, crr, mdh, icd, fliC* (conserved *5′* and *3′* ends)*, trpB, putP, aceK, mutS, trpC, tonB,* and *trpA*. Under the mutator hypothesis, the genetic distance between the pathogenic O157:H7 strain (or the closely related strain ECOR37) and the outgroup (Typhimurium) is expected to exceed the distance between the commensal K-12 and the outgroup. Prolonged periods of enhanced mutation rate should drive the points above the dotted line marking equal rates of molecular evolution. Two loci (*tonB* and *trpA*) show departure from the equal rate line, but neither has evolved differently from that expected by the molecular clock. The sequences of 12 genes were obtained from GenBank or the original sources as follows: *aceK* (5), *crr* (6-8), *fliC* (9), *gap* (10), *icd* (11), *mdh* (12), *mutS* (13,14), *putP* (15), *tonB* (16,17), *trp* (17-20).

lineages. An elevated mutation rate in O157:H7 over evolutionary time should result in greater divergence from Typhimurium than from K-12 and in the distribution of points above the equal-rate line. For both synonymous and nonsynonymous sites, most genes fall below or very near the equal-rate line with only two exceptions: *tonB* and *trpA* deviate in the direction expected under the mutator hypothesis. To test the significance of these deviations, we compared the observed degrees of divergence of K-12 and O157:H7 from Typhimurium and the expectations of the molecular evolutionary clock hypothesis (21). The basis of this test is that a constant rate of mutation results in equal numbers of substitutions in two sequences from an outgroup (21). Considering synonymous and nonsynonymous changes together with Typhimurium as an outgroup, we found that 11 of the 12 loci, including *tonB* ($m_1 = 0$, $m_2 = 3$, $X^2 = 3.00$, $p > 0.05$) and *trpA* ($m_1 = 4$, $m_2 = 10$, $X^2 = 2.57$, $p > 0.05$), did not deviate significantly from a uniform rate of evolution predicted by a molecular clock. Only *mdh* exhibited a significant departure from the molecular clock ($m_1 = 14$, $m_2 = 5$, $X^2 = 4.26$, $p < 0.05$); however, the direction was away from that predicted by the mutator hypothesis (the Typhimurium–K-12 distance exceeded the Typhimurium-O157:H7 distance).

Our findings do not conflict with the observation that MMR defects occur in relatively high frequency in emerging pathogens; however, the findings indicate no evidence of a genomewide elevation of the mutation rate in pathogenic *E. coli* O157:H7. The uniform rate of divergence of O157:H7 and K-12 suggests several possibilities. One is that the mutator state is transient and so brief that the impact on long-term rates of evolution is undetectable. This possibility is consistent with the view that mutators may generate favorable mutations in periods of intense selection and then revert to a nonmutator phenotype (22,23). Another possibility is that all bacterial populations experience brief episodes of adaptive evolution driven by hypermutation. Matic and co-workers (24) found equivalent frequencies of mutators among strains of commensal bacteria and both emerging and classical pathogenic *E. coli*.

Finally, defects in MMR that produce the mutator phenotype also relax the normal barriers to recombinational exchange between bacterial species (25). The enhanced recombination that accompanies the mutator phenotype may explain why *E. coli* O55:H7, the immediate ancestor of O157:H7 (26) that also carries the same defective MMR allele (3), harbors such an extraordinary variety of plasmid and chromosomal virulence factors (27). Together with our finding of clock-like divergence of *E. coli* O157:H7 housekeeping genes, these observations indicate that the main evolutionary benefit of the mutator phenotype is the enhanced ability to acquire useful foreign DNA (3), not an increased rate of point mutation over the long term.

Dr. Whittam is professor of biology at the Institute of Molecular Evolutionary Genetics, Pennsylvania State University. His research focuses on understanding how evolutionary forces operate to determine the amount and organization of genetic variation in natural populations of bacteria. Specific studies include the evolution of pathogenic forms of *Escherichia coli* associated with intestinal and extraintestinal infections, the evolution of virulence and resistance in a host-parasite interaction using an amoeba-*Legionella* system, and the ecologic determinants and evolution of host specificity in *Rhizobium*-legume associations.

### References

1. Riley LW, Remis RS, Helgerson SD, McGee HB, Wells JG, Davis BR, et al. Hemorrhagic colitis associated with a rare *Escherichia coli* serotype. N Engl J Med 1983;308:681-5.
2. Doyle MP, Zhao T, Meng J, Zhao S. *Escherichia coli* O157:H7. In: Doyle MP, Beuchat LR, Montville TJ, editors. Food microbiology: fundamentals and frontiers. Washington: American Society for Microbiology; 1997. p. 171-91.
3. LeClerc JE, Li B, Payne WL, Cebula TA. High mutation frequencies among *Escherichia coli* and *Salmonella* pathogens. Science 1996;274:1208-11.
4. Kumar S, Tamura K, Nei M. MEGA: molecular evolutionary genetics analysis [computer program]. Version 1.0. University Park (PA): The Pennsylvania State University; 1993.
5. Nelson K, Wang F-S, Boyd EF, Selander RK. Size and sequence polymorphism in the isocitrate dehydrogenase kinase/phosphatase gene (*aceK*) and flanking regions in *Salmonella enterica* and *Escherichia coli*. Genetics 1997;147:1509-20.
6. Hall BG, Sharp PM. Molecular population genetics of *Escherichia coli*: DNA sequence diversity at the *celC*, *crr*, and *gutB* loci of natural isolates. Mol Biol Evol 1992;9:654-65.
7. Nelson SO, Schuitema AR, Benne R, van der Ploeg LH, Plijter JS, Aan F, et al. Molecular cloning, sequencing, and expression of the *crr* gene: the structural gene for IIIGlc of the bacterial PEP:glucose phosphotransferase system. EMBOJ 1984;3:1587-93.
8. Saffen DW, Presper KA, Doering TL, Roseman S. Sugar transport by the bacterial phosphotransferase system. Molecular cloning and structural analysis of the *Escherichia coli ptsH, ptsI*, and *crr* genes. J Biol Chem 1987;262:16241-53.

9. Li J, Nelson K, McWhorter AC, Whittam TS, Selander RK. Recombinational basis of serovar diversity in *Salmonella enterica*. Proc Natl Acad Sci U S A 1994;91:2552-6.
10. Nelson K, Whittam TS, Selander RK. Nucleotide polymorphism and evolution in the glyceraldehyde-3-phosphate dehydrogenase gene (*gapA*) in natural populations of *Salmonella* and *Escherichia coli*. Proc Natl Acad Sci U S A 1991;88:6667-71.
11. Wang FS, Whittam TS, Selander RK. Evolutionary genetics of the isocitrate dehydrogenase gene (*icd*) in *Escherichia coli* and *Salmonella enterica*. J Bacteriol 1997;179:6551-9.
12. Boyd EF, Nelson K, Wang F-S, Whittam TS, Selander RK. Molecular genetic basis of allelic polymorphism in malate dehydrogenase (*mdh*) in natural populations of *Escherichia coli* and *Salmonella enterica*. Proc Natl Acad Sci U S A 1994;91:1280-4.
13. Haber LT, Pang PP, Sobell DI, Mankovich JA, Walker GC. Nucleotide sequence of the *Salmonella typhimurium mutS* gene required for mismatch repair: homology of MutS and HexA of *Streptococcus pneumoniae*. J Bacteriol 1988;170:197-202.
14. Schlensog V, Bock A. The *Escherichia coli fdv* gene probably encodes *mutS* and is located at minute 58.8 adjacent to the *hyc-hyp* gene cluster. J Bacteriol 1991;173:7414-5.
15. Nelson K, Selander RK. Evolutionary genetics of the proline permease gene (*putP*) and the control region of the proline utilization operon in populations of *Salmonella* and *Escherichia coli*. J Bacteriol 1992;174:6886-95.
16. Hannavy K, Barr GC, Dorman CJ, Adamson J, Mazengera LR, Gallagher MP, et al. TonB protein of *Salmonella typhimurium*. A model for signal transduction between membranes. J Mol Biol 1990;216:897-910.
17. Milkman R. Recombinational exchange among clonal populations. In: Neidhardt FC, Curtiss IR, Ingraham JL, Lin ECC, Low KB, Magasanik B, et al, editors. *Escherichia coli* and *Salmonella*: cellular and molecular biology. 2nd ed. Washington: American Society for Microbiology; 1996. p. 2663-84.
18. Crawford IP, Nichols BP, Yanofsky C. Nucleotide sequence of the *trpB* gene in *Escherichia coli* and *Salmonella typhimurium*. J Mol Biol 1980;142:489-502.
19. Horowitz H, Van Arsdell J, Platt T. Nucleotide sequence of the *trpD* and *trpC* genes of *Salmonella typhimurium*. J Mol Biol 1983;169:775-97.
20. Nichols BP, Yanofsky C. Nucleotide sequences of *trpA* of *Salmonella typhimurium* and *Escherichia coli*: an evolutionary comparison. Proc Natl Acad Sci U S A 1979;76:5244-8.
21. Tajima F. Simple methods for testing the molecular evolutionary clock hypothesis. Genetics 1993;135:599-607.
22. Rosenberg SM, Thulin C, Harris RS. Transient and heritable mutators in adaptive evolution in the lab and in nature. Genetics 1998;148:1559-66.
23. Taddei F, Radman M, Maynard-Smith J, Toupance B, Goupon PH, Godelle B. Role of mutator alleles in adaptive evolution. Nature 1997;387:700-2.
24. Matic I, Radman M, Taddei F, Picard B, Doit C, Bingen E, et al. Highly variable mutation rates in commensal and pathogenic *Escherichia coli*. Science 1997;277:1833-4.
25. Rayssiguier C, Thaler DS, Radman M. The barrier to recombination between *Escherichia coli* and *Salmonella typhimurium* is disrupted in mismatch-repair mutants. Nature 1989;342:396-401.
26. Whittam TS, McGraw EA, Reid SD. Pathogenic *Escherichia coli* O157:H7: a model for emerging infectious diseases. In: Krause RM, editor. Emerging infections. New York: Academic Press; 1998. p. 163-83.
27. Rodrigues J, Scaletsky ICA, Campos LC, Gomes TAT, Whittam TS, Trabulsi LR. Clonal structure and virulence factors in strains of *Escherichia coli* of the classic serogroup O55. Infect Immun 1996;64:2680-6.
